# Statistics of Suicide in France

**Published:** 1851-07-01

**Authors:** 


### STATISTICS OF SUICIDE IN FRANCE,

### FROM 1835 TO 1846, INCLUSIVE.*

At no period has so much been written, at no period so little read, as in our own days. A host of curious works and interesting documents are printed only to be forgotten—frequently most unjustly, as seems to us the case with regard to a thesis, recently published by Dr. Petit, formerly assistant resident medical officer at the Marleville Asylum, on the "*Etiology of Suicide.*" This work, in other days, would have obtained for its author the favourable notice of all who occupy themselves with social and political economy. Although undertaken as a medical study, this remarkable inquiry throws a new light on one of the social infirmities of our times, and contains instructive information for the moralist. There is no fine writing or declamation, but figures—scarcely anything but figures—which, in our opinion, speak most eloquently. Here is the proof.

The number of suicides in France in the space of twelve years, from 1st January, 1835, to 31st December, 1846, was 33,032; that is, 2,752 in each year, or one in 12,646 inhabitants. This sum was thus divided:—

| | | | | |
|---|---|---|---|---|
| Males . . . . . . . . | 24,762 | or | 2,062 | a-year. |
| Females . . . . . . . | 8,270 | or | 690 | „ |
| In twelve years . . . | 33,032 | or | 2,752 | „ |

Suicides are three times more numerous among men than among women. This proportion is almost constant in all years and in each department. The following was the number of suicides in each year, from 1835 to 1846 inclusive:

| | Males. | Females. | Total. |
|---|---|---|---|
| 1835 | 1,785 | 520 | 2,305 |
| 1836 | 1,775 | 565 | 2,340 |
| 1837 | 1,811 | 632 | 2,443 |
| 1838 | 1,886 | 700 | 2,586 |
| 1839 | 2,049 | 698 | 2,747 |
| 1840 | 2,040 | 712 | 2,752 |
| 1841 | 2,139 | 675 | 2,814 |
| 1842 | 2,129 | 737 | 2,866 |
| 1843 | 2,291 | 729 | 3,020 |
| 1844 | 2,197 | 776 | 2,973 |
| 1845 | 2,332 | 752 | 3,084 |
| 1846 | 2,329 | 773 | 3,102 |
| | 24,762 | 8,270 | 33,032 |

It will be noticed, that with the one exception of 1843, when there was a slight diminution, amounting to forty-four cases only, the number of these crimes has gone on increasing from year to year. From 2,305 in 1835, it rises to 3,102 in 1846.

The number of suicides is not equally divided in proportion to the popu-

* From an article in the "Annuaire de l'Économie Politique," 1851, by M. A. de Watteville, Inspector-General of Benevolent Societies.

lation of the various departments, but varies considerably in different localities. For the whole of France there is one suicide a-year in 12,646 inhabitants. For the department of the Seine, which counts the highest number, the proportion is one suicide in 2788 inhabitants; whilst in the lowest on the list—that of Ariége—it is only one in 84,542 inhabitants.

The following are the ten departments in which the greatest number of suicides occur:

| | In 12 years. | Per annum. | | |
|---|---|---|---|---|
| 1. Seine | 5,890 | 491 | 1 in 2,778 | inhabitants |
| 2. Seine-et-Oise | 1,200 | 100 | 1 in 4,749 | „ |
| 3. Oise | 950 | 79 | 1 in 4,984 | „ |
| 4. Seine-et-Marne | 795 | 66 | 1 in 5,139 | „ |
| 5. Marne | 795 | 66 | 1 in 5,685 | „ |
| 6. Seine (Inferior) | 1,212 | 101 | 1 in 7,577 | „ |
| 7. Aube | 404 | 34 | 1 in 7,794 | „ |
| 8. Loiret | 495 | 41 | 1 in 8,049 | „ |
| 9. Aisne | 822 | 68 | 1 in 8,123 | „ |
| 10. Var | 478 | 40 | 1 in 8,970 | „ |

With the exception of Var, all these departments are situated in the plain; whilst, on the contrary, the departments which give the smallest amount of suicides are all mountainous, excepting Gers, which is in the plain. In general, the mountainous districts have few of these crimes to deplore, as is here seen:

| | In 12 years. | Per annum. | | |
|---|---|---|---|---|
| 1. Ariége | 39 | 3 | 1 in 84,542 | inhabitants |
| 2. Aveyron | 60 | 5 | 1 in 77,824 | „ |
| 3. Corse | 48 | 4 | 1 in 57,568 | „ |
| 4. Loire (Upper) | 78 | 6 | 1 in 47,255 | „ |
| 5. Pyrenees (Upper) | 68 | 5 | 1 in 44,872 | |
| 6. Lozère | 39 | 3 | 1 in 44,791 | „ |
| 7. Gers | 94 | 8 | 1 in 40,367 | , |
| 8. Puy-de Dôme | 184 | 16 | 1 in 39,313 | |
| 9. Garonne (Upper) | 151 | 13 | 1 in 38,249 | |
| 10. Loire | 146 | 12 | 1 in 37,503 | „ |

All ages furnish their deplorable contingent of suicides, but the proportion varies at different ages. Infancy gives but few cases, youth a considerable number, middle age the most, old age next after.

| | Males. | Females. | Total. | | |
|---|---|---|---|---|---|
| Under 16 years | 183 | 56 | 239 | | |
| From 16 to 21 | 1,017 | 576 | 1,593 | 1 in 22,417 | inhabitants |
| From 21 to 30 | 3,859 | 1,375 | 5,234 | 1 in 11,443 | „ |
| From 30 to 40 | 4,508 | 1,271 | 5,839 | 1 in 10,325 | „ |
| From 40 to 50 | 5,117 | 1,558 | 6,675 | 1 in 8,078 | „ |
| From 50 to 60 | 4,010 | 1,422 | 5,432 | 1 in 8,378 | „ |
| From 60 to 70 | 3,108 | 1,076 | 4,184 | 1 in 8,125 | „ |
| From 70 to 80 | 1,575 | 546 | 2,121 | 1 in 8,717 | „ |
| Above 80 years | 344 | 114 | 458 | 1 in 10,546 | „ |
| Unknown age | 981 | 276 | 1,257 | | |
| | 24,762 | 8,270 | 33,032 | | |

The total of 239 children is composed of—

| | | | |
|---|---|---|---|
| 1 Child at | 7 years | 29 Children at | 13 years |
| 3 Children at | 8 „ | 64 „ | 14 „ |
| 2 „ | 9 „ | 75 „ | 15 „ |
| 8 „ | 10 „ | | |
| 9 „ | 11 „ | 239 | |
| 20 „ | 12 „ | | |

The predisposition to suicide goes on augmenting from the first exercise of the intellectual powers, to the period of their fullest development at about forty to fifty years of age. It remains almost stationary in old age, and does not decrease until imbecility commences.

Of the seasons of the year, winter furnishes the smallest number of suicides; the month of December, the minimum in that quarter. In January, the number begins to increase; February yields a larger proportion than January, March than February, so that the rate of increase goes on rapidly increasing up to June, when it attains its maximum. July affords a slight diminution, August a much larger decrease, the numbers diminishing progressively in inverse order to the ascending progression.

| Year. | Jan. | Feb. | Mar. | April. | May. | June. | July. | Aug. | Sept. | Oct. | Nov. | Dec. | Total. |
|---|---|---|---|---|---|---|---|---|---|---|---|---|---|
| 1835 | 160 | 152 | 205 | 201 | 239 | 241 | 294 | 219 | 169 | 158 | 162 | 105 | 2,305 |
| 1836 | 156 | 165 | 205 | 193 | 249 | 261 | 283 | 209 | 161 | 182 | 146 | 130 | 2,340 |
| 1837 | 175 | 176 | 213 | 226 | 244 | 261 | 285 | 210 | 194 | 182 | 138 | 139 | 2,443 |
| 1838 | 150 | 143 | 230 | 236 | 278 | 299 | 298 | 251 | 207 | 196 | 154 | 144 | 2,586 |
| 1839 | 172 | 185 | 228 | 251 | 304 | 275 | 296 | 238 | 218 | 207 | 194 | 179 | 2,747 |
| 1840 | 222 | 217 | 215 | 284 | 304 | 287 | 262 | 239 | 199 | 190 | 158 | 175 | 2,752 |
| 1841 | 175 | 184 | 276 | 279 | 296 | 281 | 298 | 244 | 209 | 206 | 176 | 190 | 2,814 |
| 1842 | 191 | 195 | 226 | 239 | 312 | 348 | 270 | 299 | 291 | 177 | 194 | 224 | 2,866 |
| 1843 | 225 | 230 | 283 | 258 | 318 | 334 | 336 | 267 | 207 | 194 | 198 | 170 | 3,020 |
| 1844 | 219 | 181 | 271 | 286 | 304 | 346 | 306 | 264 | 223 | 237 | 199 | 137 | 2,973 |
| 1845 | 192 | 143 | 233 | 311 | 317 | 358 | 301 | 263 | 273 | 282 | 201 | 210 | 3,084 |
| 1846 | 230 | 200 | 285 | 277 | 342 | 330 | 302 | 308 | 241 | 231 | 182 | 174 | 3,102 |
| | 2,267 | 2,171 | 2,870 | 3,041 | 3,507 | 3,621 | 3,531 | 3,014 | 2,492 | 2,442 | 2,102 | 1,977 | 33,032 |

The heat of summer is not the only and principal cause, as has been stated, of the augmentation of suicides at that season, for they begin to increase in January, and reach the maximum in June, whilst the temperature goes on mounting in July. But it is remarkable that the progressive increase and decrease in the number of suicides coincide exactly with the lengthening and shortening of the days. And it has been satisfactorily established, that only a few individuals destroy themselves during the night.

We have not sufficient data to establish with certainty the influence of occupation on suicide; for we cannot ascertain the number of persons in each employment. It would be very interesting to know whether certain employments predispose to suicide more than others; if the liberal professions, which tax the intellect, develop this propensity in a greater extent than manual labour.

PROFESSION OR OCCUPATION OF SUICIDES.

| I. | Males. | | Females. |
|---|---|---|---|
| Shepherds and shepherdesses | 188 | .. | 25 |
| Wood-cutters, charcoal-burners | 59 | .. | 6 |
| Agricultural labourers (masters and servants) | 7,530 | .. | 2,332 |
| II. | | | |
| Workers in wood | 1,109 | .. | 65 |
| „ skins, leathers, &c. | 257 | .. | 20 |
| „ iron, metals, &c. | 901 | .. | 49 |
| „ linen, cotton, silk, &c. | 894 | .. | 649 |
| Stonemasons, bricklayers, slaters, &c. | 573 | .. | 30 |
| Various occupations | 41 | .. | 55 |

| | Males. | | Females. |
|---|---|---|---|
| III. | | | |
| Bakers, pastry-cooks | 228 | .. | 21 |
| Butchers and sausage-makers | 165 | .. | 14 |
| Millers | 179 | .. | 17 |
| IV. | | | |
| Hatters | 73 | .. | 14 |
| Boot-makers | 535 | .. | 27 |
| Hair-cutters | 115 | .. | 2 |
| Tailors, upholsterers, sempstresses | 421 | .. | 522 |
| Washermen and women | 42 | .. | 148 |
| V. | | | |
| Stockbrokers | 19 | | |
| Shopkeepers | 756 | .. | 195 |
| Hawkers and hucksters | 148 | .. | 38 |
| Manufacturers, warehousemen, bankers, &c. | 293 | .. | 9 |
| Shopmen, &c. | 292 | .. | 18 |
| VI. | | | |
| Messengers, porters, water-carriers | 292 | .. | 1 |
| Mariners, watermen, bargemen, &c. | 240 | .. | 12 |
| Carriers, cabmen, coachmen, &c. | 282 | .. | 4 |
| VII. | | | |
| Hotel, inn, and coffee-house-keepers | 493 | .. | 109 |
| Household servants | 793 | .. | 832 |
| VIII. | | | |
| Artists | 118 | .. | 14 |
| Clerks, copyists | 163 | .. | 1 |
| Students | 89 | .. | 2 |
| Public officers and police | 146 | | |
| Professors and schoolmasters | 95 | | |
| Soldiers, gendarmes, veterans | 1,883 | | |
| Lawyers, medical men, other liberal professions | 300 | | |
| Persons of independent means | 1,571 | .. | 564 |
| IX. | | | |
| Rag-pickers | 19 | .. | 5 |
| Prostitutes | .. | .. | 31 |
| Beggars and vagabonds | 209 | .. | 85 |
| Uncertain professions | 658 | .. | 1,081 |
| Profession or occupation unknown | 1,689 | .. | 1,518 |
| | 24,762 | .. | 8,270 |
| Total.... | 33,032 | | |

The wish to avoid suffering exercises a considerable influence in determining the choice of the method of self-destruction, but this is somewhat counteracted by the facility and promptitude of execution in certain manners. Thus more than one-third destroyed themselves by submersion. Suspension and strangulation are selected less frequently. One-eighth perished by fire-arms. Next came asphyxia from charcoal, precipitation from elevated places, the use of cutting or penetrating instruments, and lastly poison.

The profession or occupation of an individual influences his selection of the means of death. Thus soldiers, gendarmes, and others habituated to the use of fire-arms, commonly destroy themselves with those weapons; whilst sempstresses, and particularly washerwomen, choose suffocation by means of charcoal. Nevertheless, ninety-five women killed themselves with fire-arms. It should be noticed, that four-fifths of the deaths from lighted charcoal occurred in the department of the Seine.

The presumable and probable causes of suicide are very varied and diverse. In some cases, the motives are most affecting and almost excusable; in others, foolish, trivial, and very unlikely. We will cite some examples.

| I. | Males. | Females. | Total. |
|---|---|---|---|
| Poverty, or dread of poverty | 1,464 | 389 | 1,853 |
| Debt, pecuniary embarrassment | 1,591 | 106 | 1,697 |
| Losses at play | 113 | 1 | 114 |
| Loss of situation or employment | 134 | 9 | 143 |
| Loss of law-suit | 79 | 8 | 87 |
| Pecuniary and other losses | 194 | 39 | 233 |
| Reverse of fortune not specified | 210 | 34 | 244 |
| Regret at having spent the whole or part of fortune... | 34 | 12 | 46 |
| Disappointed hope of assistance in being established in life | 43 | 8 | 51 |
| **II.** | | | |
| Grief at being exiled | 25 | ... | 25 |
| Grief at the loss of a spouse, child, or relative | 235 | 138 | 373 |
| Sorrow at the departure of children | 12 | 11 | 23 |
| Sorrow at ingratitude or misconduct of children | 88 | 47 | 135 |
| Disappointment at loss of a betrothed | ... | 1 | 1 |
| Grief at the loss of a friend or master | 7 | 1 | 8 |
| Desire to relieve family of a burden | 7 | 3 | 10 |
| „ to exempt a son from the conscription | 30 | 15 | 45 |
| Sorrow at being compelled to live apart from his family | 1 | ... | 1 |
| „ at seduction of his sister | 1 | ... | 1 |
| „ at seeing his sister abandoned by her husband | 1 | ... | 1 |
| „ at not being acknowledged by father | 2 | ... | 2 |
| „ at having no children | 1 | 1 | 2 |
| „ at misfortunes of a father | 2 | ... | 2 |
| „ at second marriage of father or mother | 2 | ... | 2 |
| „ of children ill-used or scolded by their parents | 96 | 47 | 143 |
| Disputes on money matters with parents | 81 | 17 | 98 |
| Jealousy between brothers and sisters | 17 | 7 | 24 |
| Fear of not being able to suckle her infant | ... | 1 | 1 |
| Domestic afflictions not specified | 1,909 | 714 | 2,623 |
| **III.** | | | |
| Disappointed affections | 603 | 380 | 983 |
| Jealousy between married couples or lovers | 162 | 86 | 148 |
| Pregnancy before marriage | ... | 150 | 150 |
| Remorse at having seduced a girl | 2 | ... | 2 |
| Disgust of married life | 24 | 13 | 37 |
| Imputation of paternity | 4 | ... | 4 |
| Shame or remorse for some evil act | 135 | 59 | 194 |
| Idleness | 58 | 2 | 60 |
| Misconduct, debauchery | 870 | 121 | 991 |
| Intoxication (in a fit of) | 440 | 55 | 495 |
| Habitual drunkenness | 1,162 | 198 | 1,360 |
| Remorse at having killed a person | 1 | ... | 1 |
| Vexation at being suspected of a theft | 7 | 5 | 12 |
| „ at not being able to obtain revenge | 2 | ... | 2 |
| „ at failing to pass an examination | 1 | ... | 1 |
| „ at not being able to procure some article of dress | ... | 3 | 3 |
| „ at losing some birds | 1 | ... | 1 |

| | Males. | Females. | Total. |
|---|---|---|---|
| Desire to escape the hands of justice | 970 ... | 216 ... | 1,186 |
| „ the execution of a sentence | 101 ... | 16 ... | 117 |
| „ the conscription | 13 ... | ... ... | 13 |
| „ some physical suffering | 2,032 ... | 713 ... | 2,745 |
| Suicide after assassination | 194 ... | 14 ... | 208 |
| Disgust of life | 874 ... | 183 ... | 1,057 |
| Political exaltation | 6 ... | ... ... | 6 |
| Insanity | 4,022 ... | 2,427 ... | 6,449 |
| Unknown motives | 3,015 ... | 817 ... | 3,832 |
| | 24,762 | 8,270 | 33,032 |

